# Comparison of Vocal Fold Vibratory Characteristics in Adductor Spasmodic Dysphonia and Muscle Tension Dysphonia Using High-Speed Videolaryngoscopy with 2D and Line Digital Kymography

**DOI:** 10.3390/jcm14248695

**Published:** 2025-12-08

**Authors:** Nayeon Choi, Su Na Park, GilJoon Lee

**Affiliations:** 1 Department of Otorhinolaryngology–Head and Neck Surgery, Samsung Medical Center, Sungkyunkwan University School of Medicine, Seoul 06351, Republic of Korea; 2 Department of Otorhinolaryngology–Head and Neck Surgery, School of Medicine, Kyungpook National University, Daegu 41566, Republic of Korea; 3 Department of Otorhinolaryngology–Head and Neck Surgery, Kangbuk Samsung Hospital, Sungkyunkwan University School of Medicine, Seoul 03181, Republic of Korea

**Keywords:** spasmodic dysphonia, muscle tension dysphonia, high-speed videolaryngoscopy, digital kymography, phase symmetry, amplitude symmetry, oscillatory break, supraglottic hyperfunction

## Abstract

**Background/Objectives:** Differentiating adductor spasmodic dysphonia (AdSD) from muscle tension dysphonia (MTD) is challenging because their auditory–perceptual features often overlap. We examined whether high-speed videolaryngoscopy (HSV) combined with two-dimensional digital kymography (2D-DKG) and line DKG yields qualitative signs and quantitative metrics that distinguish AdSD from MTD. **Methods:** We analyzed vocal fold vibration in eight patients with AdSD, eight with primary MTD, and eleven vocally healthy controls using a multifunctional system integrating HSV, 2D-DKG, and line DKG. Qualitative features (glottal closure, mucosal wave, phase and amplitude symmetry, oscillatory breaks, and supraglottic hyperfunction) and quantitative indices (closed quotient [CQ], speed quotient [SQ], phase symmetry index [PSI], amplitude symmetry index [ASI]) were assessed. Group differences were tested with one-way ANOVA and Scheffé post hoc comparisons. **Results:** Oscillatory breaks were observed in 75% of AdSD cases and in 0% of MTD and controls, whereas supraglottic hyperfunction occurred in 100% of MTD and in 0% of AdSD and controls. Quantitatively, CQ, SQ, PSI, and ASI differed between dysphonic groups and controls (*p* < 0.05), but no quantitative index discriminated against AdSD from MTD. **Conclusions:** HSV with 2D-/line-DKG provides complementary, objective information on vibratory patterns and supraglottic behavior, supporting targeted qualitative assessment in the clinical differentiation between AdSD and MTD, and highlighting the need for its incorporation into clinical practice as a procedure to assist in the complex diagnostic distinction between these conditions.

## 1. Introduction

The exact differential diagnosis between adductor spasmodic dysphonia (AdSD) and muscle tension dysphonia (MTD) has long been debated [1,2,3,4,5]. Although AdSD is not clearly identified, it is a neurological voice disorder caused by the involuntary contraction of the vocal folds [6]. MTD is a functional (=behavioral) voice disorder caused by excessive muscle tension without structural abnormalities around the larynx [7]. The two conditions share overlapping features—such as a strained or pressed voice, voice breaks, and phonatory interruptions—yet their etiologies and treatments differ, making accurate differential diagnosis essential.

For decades, investigators have compared the two disorders using a variety of voice assessments [1,2,8,9,10]. With accumulating evidence, many have concluded that laryngeal endoscopy or stroboscopy and auditory–perceptual evaluation often provide greater diagnostic value than purely acoustic or aerodynamic measures. This view is consistent with Titze’s classification of voice signals into three types, which recommends selecting analytic methods according to signal periodicity [11]. Type 1 voices (periodic or near-periodic) are amenable to acoustic perturbation measures such as jitter and shimmer; type 2 voices (signals with subharmonics or bifurcations) and type 3 voices (aperiodic or irregular signals) are better evaluated using visual displays and auditory–perceptual analysis. Because AdSD and MTD frequently deviate from strict periodicity, they often correspond to type 2 or type 3 signals under Titze’s framework; thus, visual or auditory–perceptual approaches are appropriate. Nevertheless, such evaluations may vary with examiner expertise, underscoring the need for objective, quantitative methods to complement subjective assessment and support diagnostic validity. Both conditions share similar characteristics, such as a tense or strained voice, vocal effort, voice breaks, and interruptions in phonation, and often exhibit fluctuations in these aspects throughout the day or in different communicative contexts. In these cases, etiologies and treatments differ, which makes accuracy in differential diagnosis essential.

Traditionally, laryngeal stroboscopy has been indispensable for evaluating vocal fold vibration patterns in voice disorders. Clinically, it is valuable for detecting structural lesions and functional abnormalities. However, stroboscopy does not depict true cycle-to-cycle motion; rather, it reconstructs an apparent “slow-motion” image based on assumptions of periodicity. Consequently, it performs poorly when signals are aperiodic or unstable, as in many patients with AdSD or MTD [12,13]. In contrast, high-speed videolaryngoscopy (HSV) is independent of vibratory regularity and can record at up to 2000 frames per second, capturing actual vocal fold vibration without inter-frame aliasing. HSV data can be post-processed to generate digital kymography (DKG), enabling quantification of features beyond those visible in stroboscopy.

Recently, Wang et al. developed a multifunctional laryngeal performance testing system that integrates HSV with line-scanning DKG (line DKG) and plane-scanning, two-dimensional DKG (2D-DKG), allowing simultaneous visualization of three complementary outputs on a single monitor [14,15]. In line DKG, a scan line is positioned across selected points of the glottis to display the temporal evolution of vibratory motion [16]. From these data, parameters such as open quotient (OQ), closed quotient (CQ), speed quotient (SQ), phase symmetry index (PSI), and amplitude symmetry index (ASI) can be derived. The addition of 2D-DKG facilitates appraisal of the overall spatiotemporal vibratory pattern. Collectively, these techniques provide objective metrics for analysis and interpretation grounded in laryngeal anatomy and pathology. Their utility has been demonstrated in healthy adults and in various voice disorders, and they are well-suited for challenging differential diagnoses such as AdSD versus MTD [14,15,16].

We hypothesized that qualitative phenomena captured on HSV/DKG—such as oscillatory breaks and supraglottic hyperfunction—would discriminate AdSD from MTD more robustly than commonly used quantitative measures (CQ, SQ, PSI, ASI) and tested this using an integrated HSV–2D/line DKG system.

## 2. Materials and Methods

### 2.1. Enrolled Patients

The subjects of this study were eight patients with AdSD (8 females, 51.38 ± 1.40 years old) and eight MTD (6 females, 2 males, 38.13 ± 1.12 years old) diagnosed by an otolaryngologist. AdSD was targeted for those who responded through Botox injection, and those with negative tremors were excluded. MTD was primary, and people who had no experience with Botox injection or medication improved through voice therapy. The voice data for them used the data obtained from the initial evaluation. Two voice language therapists with more than 5 years of voice disorder experience listened to their voices and independently assessed the GRBAS scale and voice discontinuity. In common, they were those who scored between 1 and 2 and generally exhibited a hoarse voice, effortful voice, or voice interruptions. The control group consisted of people who were matched for age and gender and evaluated as having a normal voice on the GRBAS scale, excluding those with voice problems (10 females, 1 male, 48.23 ± 2.32 years old. Participants were categorized into three groups: (1) an AdSD group comprising patients diagnosed with AdSD, (2) an MTD group comprising patients diagnosed with primary muscle tension dysphonia as a functional (behavioral) voice disorder, and (3) a control (vocally healthy) group consisting of individuals with no history of voice complaints or laryngeal pathology and with normal laryngeal findings on examination. For clarity, these three groups are hereafter referred to as the AdSD group, the MTD group, and the control (vocally healthy) group, respectively.

### 2.2. Diagnosis of Muscle Tension Dysphonia and Adductor Spasmodic Dysphonia

Diagnoses of AdSD and muscle tension dysphonia (MTD) were established in a multidisciplinary voice clinic by an experienced laryngologist and a certified speech-language pathologist, based on consensus. For AdSD, the diagnosis was made according to standardized clinical criteria, including a history of task-specific strained or strangled voice quality with intermittent voice breaks during connected speech and endoscopic evidence of inappropriate hyperadduction of the vocal folds during phonation, in the absence of structural lesions. For MTD, the diagnosis required a persistent strained or pressed voice quality with excessive supraglottic compression on laryngoscopic examination, without evidence of organic or neurologic laryngeal pathology, and consistent with a functional (behavioral) voice disorder. All patients were evaluated using the same standardized diagnostic procedures routinely applied in our tertiary voice center.

### 2.3. Examination System and Instrument

**Figure 1:**
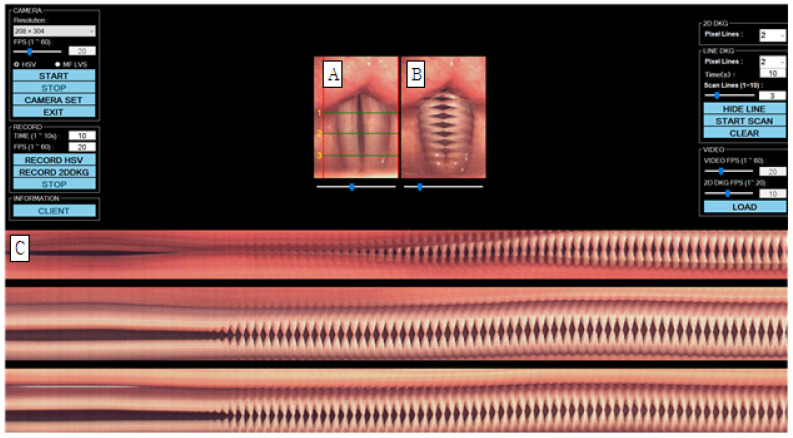
Images of vocal fold vibrations in normal 1 were obtained via multiple analyses of HSV images. (**A**) HSV, (**B**) 2D DKG, and (**C**) line DKG. HSV = high-speed videoendoscopy, 2D DKG = two-dimensional digital kymography.

Vocal fold vibration was tested using a multifunctional examination system (USC-700MF, U-Medical, Pusan, Republic of Korea) (Figure 1). The vocal folds were photographed with a 70-degree rigid endoscope (8700 CKA, Storz, Germany) with a 300 W xenon light source, and the resolution was set to 208 × 304 pixels, with the speed set to 1500 FPS. When the stored high-speed video is played back (A), a 2D DKG is created in real time (B), and a line DKG in a specific area is created after post-processing (C). For image evaluation of line DKG and 2D DKG, the pixel line was set to 213. In order to examine the vocal fold vibration characteristics, lines were created by dividing the vocal folds into three parts. Line 1 was located at the back of the vocal folds, line 2 at the center of the vocal folds, and line 3 at the front of the vocal folds. Subjects were asked to utter the vowel ‘i’ at a comfortable pitch and intensity. Because extended vocalization can be difficult, participants were instructed to speak with a 2-s break [17], and a total of 10 s of vocalization was stored.

### 2.4. Analysis of Data

(1) Qualitative analysis of vocal fold vibration through HSV, 2D-DKG, and line DKG

Indices used in previous studies were used for qualitative evaluation of vocal fold vibration. [16,17,18] Through HSV, 2D-DKG, and line DKG, glottal closure, mucosal wave, the Phase Contrast (Phase Symmetry) Index (PSI), the Amplitude Phase Index (ASI), oscillatory breaks characteristic of AdSD, and hyperfunction characteristic of MTD were additionally examined.

**Figure 2:**
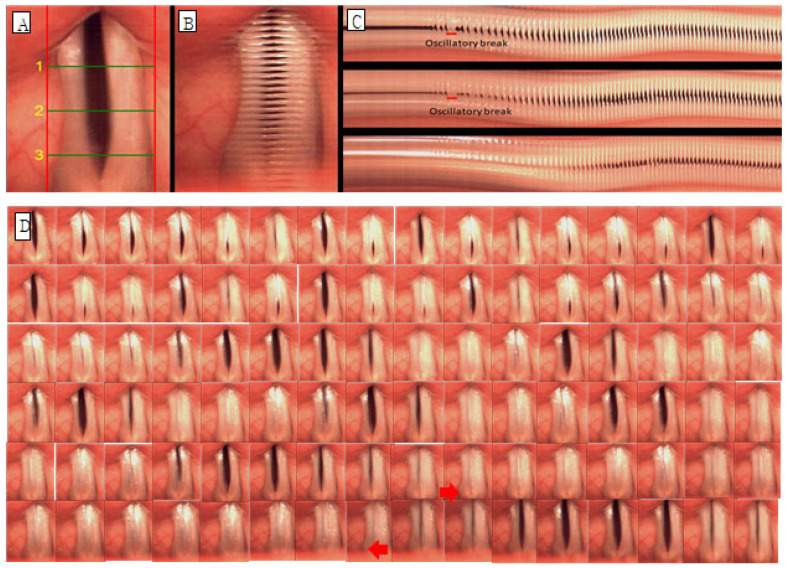
Images of vocal fold vibrations in a patient with AdSD 1 obtained via multiple analyses of HSV images. (**A**) HSV, (**B**) 2D DKG, and (**C**) line DKG. (**D**) Glottal cycle montages of oscillatory break (arrow) in the sample with adductor spasmodic dysphonia show vocal folds in a completely closed position.

**Figure 3:**
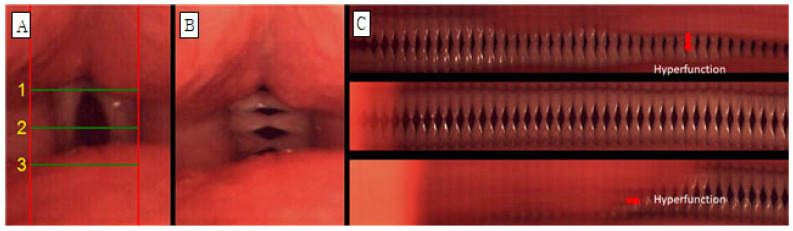
Images of vocal fold vibrations in a patient with MTD 1 obtained via multiple analyses of HSV images. (**A**) HSV, (**B**) 2D DKG, and (**C**) line DKG. Hyperfunction in line DKG.

Glottic occlusion was classified as either complete or incomplete, and all cases that were not completely closed were evaluated as incomplete. The mucosal wave was analyzed by dividing it into left and right sides. When the normal mucosal wave was about 50% of the vocal fold width, if the mucosal wave was smaller than that, it was evaluated as decreased, and if it was larger, it was increased. The phase difference is the ratio of symmetrical movement time when both vocal folds are adducted and abducted. It is evaluated as symmetrical when symmetrical and asymmetrical when not. Amplitude difference is the ratio of mucosal wave size when both vocal folds adduct and abduct, and it was evaluated as symmetrical when it was symmetrical and asymmetrical when it was not. Vibration deviation was evaluated by observing and not observing vibration deviation with complete suspension of the vocal folds in prolonged vowel phonation. Hypertonia was defined as when part of the true vocal fold was not visible due to contraction of the upper glottis, and hypertonia was evaluated as observed or not observed. For example, in the case of Figure 2, glottal closure is completely closed (B, C), mucosal waves are increased because mucosal waves are observed at 50% or more of the width of both vocal folds (B), and the phase difference is maximum for both vocal folds. There is a phase difference because the time of abduction is not the same (B, C), and there is a difference in amplitude because the magnitude of the mucosal wave between the vocal folds is different (B, C). Vibration deviation was observed (C), whereas hypertension was not observed. C in Figure 3 is the form of hypertension observed on line DKG.

(2) Quantitative analysis of vocal fold vibration through 2D DKG

**Figure 4:**
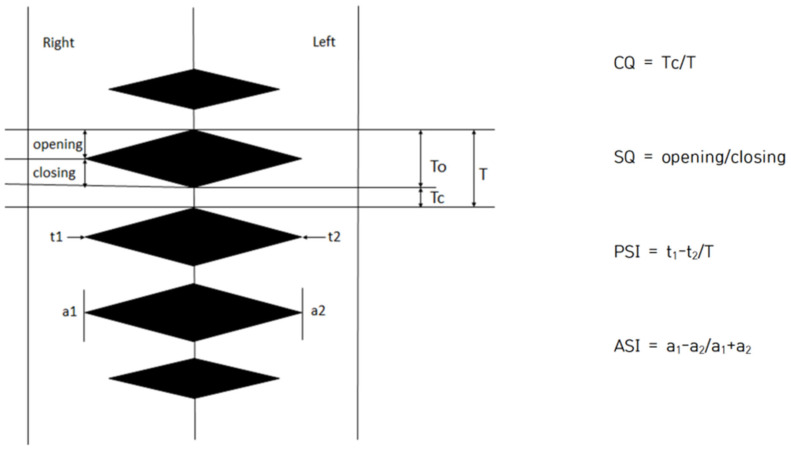
Quantitative analysis of vocal fold vibrations in 2D DKG. CQ = Closed quotient, SQ = Speed quotient, PSI = Phase symmetry index, and ASI = Amplitude symmetry index.

Image J software 1.54 (NIH, Bethesda, MD, USA) was used to analyze objective indicators of vocal fold vibration in 2D DKG images, all of which were performed manually. Referring to previous studies [12,16,18], four parameters: Closed Quotient (CQ), Speed Quotient (SQ), Phase Symmetry Index (PSI), and Amplitude Symmetry Index (ASI) were used to examine the characteristics of vocal fold vibration. CQ is the rate of time during which the glottis is closed in the vocal fold vibration cycle, and the value increases when the closing period is longer than the opening period. It is calculated by dividing by the closing time. That is, the faster the vocal folds close and the slower they open, the higher the value. The PSI is the time difference between adduction and abduction of both vocal folds, and is the value obtained by dividing the total vocal fold vibration period by the phase difference between the two vocal folds. ASI is a value obtained by dividing the sum of the amplitudes of the two vocal folds by the difference in the amplitude of the two vocal folds. PSI and ASI are in the range of −1 to 1, and the closer to 0, the more symmetrical. The reason why the values range from negative to positive is the difference depending on the direction of the right and left vocal folds. In this study, negative values were converted into absolute values because the purpose of this study was to measure the rate of vocal fold movement rather than to analyze the movement of the vocal folds in different directions. The average value was calculated by checking the indicators for each part corresponding to the three lines (back of the vocal folds, center of the vocal folds, and front of the vocal folds). This is shown in Figure 4.

### 2.5. Statistical Analysis

In addition to the researcher, a voice language rehabilitation specialist with more than 5 years of experience in voice disorder evaluation and treatment was selected for vocal fold vibration evaluation. Evaluators initially evaluated the qualitative and quantitative analysis of vocal fold vibration, and re-evaluated them 4 weeks later due to the learning effect [16]. In the case of qualitative analysis, the evaluation results were confirmed and agreed upon among the evaluators. In the case of quantitative analysis, the author quantified all parameters, and then the other evaluators confirmed the results. The resulting value was calculated. For statistical analysis, descriptive statistics (means ± standard deviations) were calculated for each quantitative parameter (CQ, SQ, PSI, and ASI) in the AdSD, MTD, and control (vocally healthy) groups. Group differences in these parameters were evaluated using one-way analysis of variance (ANOVA), followed by Scheffé’s post hoc test for pairwise comparisons between groups. All analyses were performed using SPSS version 22.0 (IBM Corp., Armonk, NY, USA), and a two-sided *p*-value < 0.05 was considered statistically significant.

## 3. Results

### 3.1. Qualitative Analysis of Vocal Fold Vibration Through HSV, 2D-DKG, and Line DKG

Table 1 shows the qualitative evaluation of vocal fold vibration in AdSD and MTD. For glottal obstruction, complete obstruction was observed in 62.5% (5/8) of AdSD and 75% (6/8) of MTD. In AdSD, mucosal perturbations were reduced on both sides in 50% (4/8) of cases, increased in 12% (1/8), and showed a difference between the two amplitudes in 37% (3/8). In MTD, amplitudes increased on both sides in 37% (3/8) of cases, decreased in 25% (2/8), and were normal in 12% (1/8). A difference between the two amplitudes was observed in 25% (2/8) of the cases. As for the phase difference, 87% (7/8) of AdSD was asymmetric and 12% (1/8) symmetric, and MTD was 75% (6/8) asymmetric and 25% (2/8) symmetric. The amplitude difference between AdSD and MTD was 87% (7/8) asymmetric and 12% (1/8) symmetric. Vocal fold vibration deviation was observed in 75% (6/8) of AdSD and not in 25% (2/8), and no vocal fold vibration deviation was observed in MTD. Hypertension was not observed in AdSD, and 100% (8/8) hypertonia was observed in MTD. In the case of the normal negative group, complete obstruction was observed in 72% (8/11), mucosal wave was normal in 82% (9/11), and increased in 18% (2/11). PSI and ASI were observed in 91% (10/11) were symmetric, and vibrational deviation and hypertension were not observed.

### 3.2. Quantitative Analysis of Vocal Fold Vibration Through 2D DKG

The descriptive values of each parameter for the AdSD, MTD, and control (vocally healthy) groups are summarized in Table 2, and the results of the statistical comparisons among the three groups are presented in Table 3. The descriptive statistics of CQ, SQ, PSI, and ASI among the three groups are presented in Table 2, and the results of one-way ANOVA are presented in Table 3. As a result of the statistical significance test, significant differences were observed in all indicators. CQ (F(2, 0.219) = 15.394, *p* < 0.001), SQ (F(2, 3.500) = 17.436, *p* < 0.01), PSI (F(2, 0.451) = 10.229, *p* < 0.01) ASI (F(2, 0.149) = 17.136, *p* < 0.001). As a result of Scheffe’s post hoc test for the values showing significant differences, there was a significant difference between AdSD and the normal-negative group (*p* = 0.000) and between MTD and the normal-negative group (*p* = 0.006) in CQ. In SQ, a significant difference was only found between AdSD and the normal-negative group. There was a significant difference between the normal-negative group (*p* = 0.000) and between the MTD and the normal-negative groups (*p* = 0.000). In PSI, there were significant differences between AdSD and the normal-negative group (*p* = 0.005) and between MTD and the normal-negative group (*p* = 0.002). In ASI, there were significant differences between AdSD and the normal-negative group (*p* = 0.000), MTD, and the normal-negative group. There was a significant difference between the normal-negative groups (*p* = 0.004). Significant differences were observed between the dysphonic and normal voice groups in all indicators, whereas no significant differences were observed between AdSD and MTD.

## 4. Discussion

Directly checking the vibration patterns of the vocal fold mucosa is the most accurate way to evaluate voice disorders [13]. Existing laryngeal stroboscopy converts vocal fold vibration into a stationary or slowly moving state, creating an optical illusion that is visible to us. Although it was a test, HSV made it possible to visually check the actual movement of the vocal folds. Recently, a multifunctional laryngeal performance test system that can simultaneously utilize 2D DKG and line DKG based on HSV has been developed, enabling voice disorders to be evaluated from a wider perspective. As the effectiveness of this device has been revealed in several previous studies [12,16,19,20,21], this study revealed differences through objective indicators and qualitative analysis for AdSD and MTD, which are difficult to differentiate in clinical practice. Notably, in this study, although the quantitative parameters (CQ, SQ, PSI, ASI) clearly differentiated the dysphonic groups from the control (vocally healthy) group, they did not reliably separate AdSD from MTD. This likely reflects the inherent overlap between these two disorders: both are characterized by strained, effortful phonation with excessive glottal closure and supraglottic hyperfunction, and our HSV-based 2D-/line-DKG measures appear to capture this shared hyperfunctional pattern more strongly than the subtle differences in their underlying pathophysiology. In addition, the limited sample size, inter-individual variability, and the use of sustained vowel phonation with a rigid endoscope, rather than connected speech or task-specific phonation, may have reduced the sensitivity of these metrics to detect finer distinctions between AdSD and MTD. Thus, the present quantitative findings should be interpreted as reflecting partially overlapping vibratory profiles rather than distinct diagnostic signatures, and they support the use of HSV-based 2D-/line-DKG as a complement—not a replacement—to qualitative assessment and clinical diagnosis.

Previous studies have used videokymography and digital kymography to derive objective measures of vocal fold vibration. Manfredi et al. proposed automated extraction of kymographic parameters from videokymographic images to characterize vibratory patterns in normal and disordered voices, demonstrating the feasibility of quantitative analysis based on line-scan images [22]. Our 2D-/line-DKG parameters (CQ, SQ, PSI, and ASI), obtained from HSV, extend this approach by providing a descriptive profile of both cycle-to-cycle irregularity and supraglottic activity across three distinct groups (AdSD, MTD, and control), thereby allowing direct comparison between a neurogenic dystonia and a functional (behavioral) voice disorder.

Chen et al. used digital kymography in vocally healthy adults and showed that normal phonation is characterized by highly periodic vibration with a dominant fundamental component and relatively low variability across cycles [23]. Consistent with their findings, our control (vocally healthy) group exhibited low CQ and SQ values and minimal supraglottic compression, providing a normative reference against which the pathological patterns in AdSD and MTD could be contrasted. In contrast, the elevated CQ, SQ, and ASI values in the AdSD and MTD groups reflect increased irregularity and hyperfunctional supraglottic behavior, highlighting the potential of HSV-based 2D-/line-DKG analysis to capture disease-specific deviations from normal vibratory patterns.

More recently, Yousef et al. applied high-speed videoendoscopy during connected speech in AdSD and used a convolutional neural network to detect frames in which the vocal folds were optically obstructed, underscoring the value of HSV for analyzing dynamic laryngeal behavior in ecologically valid speech tasks [24]. While our study focused on sustained phonation of the vowel ‘i’ using rigid HSV, the present descriptive analysis complements this line of research by providing a detailed characterization of vibratory and supraglottic patterns in AdSD and MTD that can serve as a basis for future automated or machine-learning–based approaches. Together, these findings suggest that HSV with 2D-/line-DKG can bridge the gap between established kymographic metrics and emerging data-driven methods and may ultimately assist clinicians in the complex differential diagnosis between AdSD and MTD.

Vibrational deviation, which is a characteristic of AdSD mentioned above, was also observed in this study. However, it did not appear in all subjects. Only six out of eight patients were observed, and the other two were not observed; none were observed in the MTD group or the normal negative group. Similar results were also found in previous studies. Patel et al. [17] used high-speed digital imaging (HSDI) to determine AdSD and MTD using four parameters: vibration deviation, micromotion, motion irregularities, and hyperfunction. As a result, only four out of eleven AdSD had 2–4 vibrational deviations, while none were observed in MTD. Vibrational deviation was an index observed only in AdSD but was not statistically significant in distinguishing between AdSD and MTD, and was interpreted as having low sensitivity instead of high specificity. In this study, vibrational deviation was observed only in AdSD, as in many previous studies, [17,25,26], but vibrational deviation did not necessarily appear in AdSD. However, there was no case of vibrational deviation in the normal voice group or the MTD with similar symptoms. Therefore, it can be understood that the index of vibrational deviation is characteristic of AdSD but has high specificity rather than high sensitivity. In other words, it can reduce the possibility of mistaking MTD for AdSD.

Hypertonia was a typical feature of MTD. In this study, hypertonia was observed in all MTD subjects, but no hypertonia was observed in AdSD. In the MTD, contraction of the upper glottis was observed before vocal fold vibration started, or contraction of the false glottis or upper glottis was observed in the middle of stable vocalization. Patel et al. [17] stated that hypertonia is a characteristic of MTD and a parameter that can differentiate between AdSD and MTD. This can be interpreted in the same context as the results of this study.

Complete (62.5%) and incomplete (37.5%) glottal occlusions were found evenly in AdSD. Patel et al. [17], who studied the pattern of glottal obstruction in AdSD and reported three types of glottal obstruction: complete obstruction, incomplete obstruction, and complete obstruction with pseudoglandular contact, whereas in the study of Tsuji et al. [26] only a complete glottal obstruction was observed. In the present study and in the study by Patel et al. [17] various aspects of glottal obstruction were observed, whereas in the study by Tsuji et al. [26] it was interpreted as a complete glottal obstruction due to the difference in the number of subjects. In this study and in the study by Patel et al. [17], the number of subjects was eight and eleven, whereas in Tsuji et al. [26] there was only one subject, and complete obstruction was observed in one, so the glottal obstruction pattern of AdSD was considered a complete obstruction. In this study, the posterior gap type was observed among complete obstruction and incomplete obstruction. Choi and Choi [18], who studied the pattern of glottal obstruction in normal adult women, reported that the posterior gap type was the most frequent at 54%, followed by the complete obstruction and hourglass type. In the case of men, the complete obstruction type was the most common, whereas in women, the posterior gap type was prominent. Incomplete obstruction was observed in two out of eight patients at MTD, and the type of obstruction was the posterior gap type. This may also reflect that women tend to have a wider glottal gap behind the vocal folds than men.

In PSI, AdSD, and MTD showed a significant difference from the normal negative group, but no difference was observed between the two voice disorders. PSI appears because the frequency of vocal fold vibration differs depending on the tension imbalance of the vocal folds on both sides, the difference in height, the degree of opening of the glottis immediately before vocalization, and the increase in the mass of the mucous membrane. It can be said that excessive adduction of the vocal folds hinders the airflow passing between the glottis, causing an imbalance in the tension of the vocal folds on both sides. Alternatively, a phase difference in vocal fold vibration may have occurred because one or both vocal folds contracted and did not engage normally, rather than undergoing regular abduction during vocal fold vibration.

According to the body-cover theory, the mucosal wave begins to abduct as the subglottic pressure increases below the vocal folds and opens up to the upper part of the vocal folds, forming vertically according to the stiffness of the vocal fold layer. It is predicted that the vocal fold tension imbalance between AdSD and MTD will also affect the size of the mucosal wave. A score of (3/8) was the most common. Although it can be assumed that the decrease in mucosal waves in AdSD was caused by an increase in the tension of the vocal folds, it is unreasonable to interpret this as a meaningful difference because both groups had an increase and a decrease in mucosal waves and a difference in amplitude on both sides.

The difference in mucosal waves of both vocal folds is related to ASI, but in the qualitative analysis of this study, the subjects with asymmetrical amplitude difference did not necessarily have a difference in mucosal waves. The reason for this is that when the mucosal wave was analyzed, it was determined only as a decrease or an increase based on 50%, and it was not subdivided to determine whether the vocal folds vibrated at 20% or 40% of the width of the vocal folds. Since both vocal folds belonged to the ‘decreased’ group, the difference in amplitude was analyzed separately, whether they vibrated at 20% or 40%, so even a slight difference was evaluated as amplitude asymmetry. Therefore, not all of the subjects evaluated as asymmetric had differences in mucosal waves due to amplitude differences. Most of the relatively normal voice group had 50% mucosal waves, and the phase and amplitude differences were symmetrical.

AdSD and MTD showed significantly higher CQ values than the control (vocally healthy) group, whereas no significant difference was observed between the two dysphonic groups. CQ reflects the relative duration of vocal fold contact within each vibratory cycle. In our qualitative ratings, complete glottal closure was observed in 62.5% (5/8) of the AdSD group, 75.0% (6/8) of the MTD group, and 72.7% (8/11) of the control group, suggesting no obvious group difference when closure is treated as a simple dichotomous feature. However, CQ was still significantly higher in AdSD and MTD than in controls, indicating that, beyond the mere presence of complete closure, the closed phase occupies a larger portion of the cycle in the dysphonic groups. This finding is consistent with a tension-type phonatory pattern, in which excessive activation of the vocal fold adductor muscles produces strong glottal closure and a prolonged contact phase.

In contrast, SQ values in AdSD and MTD were significantly lower than those in the control (vocally healthy) group. A higher SQ indicates that the period during which the vocal fold contact area decreases (de-contact phase) is relatively longer than the period during which it increases (contact phase), which is associated with more efficient airflow release and clearer glottal sound production. The reduced SQ observed in AdSD and MTD, therefore, suggests a relatively prolonged contact phase and shortened de-contact phase, implying that the vocal fold mucosa does not vibrate smoothly and that effective phonation is compromised. As expected, the speed-related index was significantly smaller than in the control group, in line with the perceptual impression of a strained, effortful voice quality in both disorders.

In this study, we tried to find out the difference between AdSD and MTD through HSV, 2D DKG, and line DKG. It would have been difficult to identify vocal fold vibration indices, such as very small movements of vocal fold vibration, vibration deviation, inner/outer boundary, or closure index of vocal folds, using laryngeal stroboscopy or other voice evaluation devices. However, by using HSV, 2D DKG, and line DKG, it was possible to confirm the overall vocal fold movements of AdSD and MTD, which are difficult to differentiate clinically, and to observe the changes in specific vocal fold regions over time. As a result, it was possible to evaluate the vocal fold vibration patterns from various angles, and qualitative and quantitative evaluations were possible. Among the vocal fold vibration indices such as CQ, SQ, PSI, and ASI, there was no factor that could differentiate between AdSD and MTD, but it was confirmed that vibration deviation was a characteristic observed in AdSD and hypertonicity was a characteristic observed in MTD. However, since the study was conducted with a small number of subjects, caution is required in interpretation. In addition, when selecting subjects for AdSD, except for tremors, which are a representative symptom, only those with symptoms phonetically similar to MTD were selected, which is difficult to see as a representative group of AdSD. Therefore, in future studies, it would be meaningful to compare the essential tremor group and the AdSD group, in which voice tremors are common.

This study has several limitations. First, the sample size was relatively small and consisted of patients treated at tertiary centers in a single center, which may limit the generalizability of our results to broader clinical populations. In addition, the small number of participants inevitably reduces the statistical power to detect more subtle between-group differences, so our results should be interpreted as preliminary and hypothesis-generating. Larger, multicenter studies are warranted to confirm the present findings and improve their generalizability. Second, we analyzed sustained phonation of the vowel ‘i’ only, rather than connected speech or task-specific phonation, so vibratory characteristics that emerge during more complex or conversational tasks may not have been captured. Third, quantitative parameters were derived from manual frame-by-frame measurements of 2D-DKG images, which are inherently vulnerable to tester-dependent measurement error despite consensus procedures. Fourth, the AdSD group was restricted to patients with botulinum toxin–responsive, non-tremorous AdSD, whereas the MTD group comprised primary MTD that improved with voice therapy; therefore, our findings may not be directly applicable to patients with coexisting vocal tremor, mixed spasmodic dysphonia, or refractory MTD. Finally, the retrospective, cross-sectional design precludes assessment of longitudinal changes or treatment-related effects. Future prospective studies with larger and more heterogeneous cohorts, automated or semi-automated HSV/DKG analyses, and task-specific voice assessments are needed to validate and extend these observations.

The significance of this study lies in examining and understanding the characteristics of AdSD and MTD with only vocal fold vibration patterns through HSV, 2D DKG, and line DKG.

## 5. Conclusions

This study used high-speed videoendoscopy with 2D-/line-digital kymography to describe vocal fold vibration and supraglottic activity in patients with AdSD, MTD, and vocally healthy controls. AdSD and MTD showed abnormal and partly distinct patterns compared with controls, indicating that HSV-based 2D-/line-DKG can provide useful complementary information to routine laryngoscopic and perceptual assessment. These findings suggest that this method may support the differential diagnosis between AdSD and MTD, although it should not be used as a stand-alone diagnostic tool. Clinically, qualitative HSV with 2D-/line-DKG may be incorporated as a complementary tool in routine voice assessment to support differential diagnosis between AdSD and MTD and to assist in individualized voice therapy planning. Further prospective studies with larger samples and task-specific phonation are needed to confirm and extend these results.

## Figures and Tables

**Table 1:** Descriptive analysis of the qualitative assessment of vocal fold vibration in patients with adductor spasmodic dysphonia and muscle tension dysphonia.

Group	Case	Glottal Closure	Mucosal Wave (Left)	Mucosal Wave (Right)	PSI	ASI	Oscillatory Break	Hyperfunction
**AdSD**	1	Incomplete	Decreased	Decreased	Asymmetry	Asymmetry	Absence	Absence
	2	Complete	Increased	Decreased	Asymmetry	Asymmetry	Absence	Absence
	3	Complete	Increased	Decreased	Asymmetry	Asymmetry	Absence	Absence
	4	Complete	Decreased	Decreased	Asymmetry	Asymmetry	Presence	Absence
	5	Complete	Increased	Increased	Asymmetry	Asymmetry	Absence	Absence
	6	Complete	Normal	Decreased	Asymmetry	Asymmetry	Presence	Absence
	7	Incomplete	Decreased	Decreased	Symmetry	Asymmetry	Presence	Absence
	8	Incomplete	Decreased	Normal	Asymmetry	Asymmetry	Presence	Absence
**MTD**	1	Complete	Increased	Increased	Asymmetry	Asymmetry	Absence	Presence
	2	Incomplete	Decreased	Decreased	Symmetry	Asymmetry	Absence	Presence
	3	Complete	Increased	Increased	Symmetry	Asymmetry	Absence	Presence
	4	Complete	Increased	Normal	Asymmetry	Asymmetry	Absence	Presence
	5	Incomplete	Decreased	Decreased	Asymmetry	Asymmetry	Absence	Presence
	6	Complete	Increased	Increased	Asymmetry	Asymmetry	Absence	Presence
	7	Complete	Increased	Normal	Asymmetry	Asymmetry	Absence	Presence
	8	Complete	Normal	Normal	Asymmetry	Asymmetry	Absence	Presence

**Abbreviations:** PSI = Phase Symmetry Index; ASI = Amplitude Symmetry Index; AdSD = Adductor Spasmodic Dysphonia; MTD = Muscle Tension Dysphonia.

**Table 2:** Descriptive statistics of vocal fold vibration parameters in patients with AdSD, MTD, and controls.

Parameter	MTD (N = 8)	AdSD (N = 8)	Normal (N = 11)
CQ	0.58 (0.05)	0.64 (0.08)	0.48 (0.04)
SQ	0.92 (0.31)	0.92 (0.13)	1.49 (0.24)
PSI	0.26 (0.06)	0.25 (0.16)	0.07 (0.05)
ASI	0.14 (0.05)	0.18 (0.05)	0.08 (0.04)

**Abbreviations:** CQ = Closed Quotient; SQ = Speed Quotient; PSI = Phase Symmetry Index; ASI = Amplitude Symmetry Index; CQ represents the proportion of the glottal cycle during which the vocal folds are closed; SQ is the ratio of the opening to closing duration of the glottal cycle; PSI quantifies the degree of temporal symmetry between the left and right vocal fold movements, and ASI represents the amplitude symmetry between them.

**Table 3:** Analysis of vocal fold vibration parameters in patients with AdSD, MTD, and controls.

Parameter	Source	Sum of Squares	df	Mean Square	F	*p*-Value
CQ	SSB	0.123	2	0.062	15.394	0.000 *
	SSW	0.096	24	0.004		
	SST	0.219	26			
SQ	SSB	2.073	2	1.037	17.436	0.000 *
	SSW	1.427	24	0.059		
	SST	3.500	26			
PSI	SSB	0.208	2	0.104	10.229	0.001 *
	SSW	0.244	24	0.010		
	SST	0.451	26			
ASI	SSB	0.088	2	0.044	17.136	0.000 *
	SSW	0.061	24	0.003		
	SST	0.149	26			

**Abbreviations:** CQ = Closed Quotient; SQ = Speed Quotient; PSI = Phase Symmetry Index; ASI = Amplitude Symmetry Index; SSB = Sum of Squares Between Groups; SSW = Sum of Squares Within Groups; SST = Total Sum of Squares; df = degrees of freedom. Significant differences were observed in all parameters (*p* < 0.01). Data are presented as mean ± SD. *p*-values were obtained using one-way ANOVA; * *p* < 0.05 was considered statistically significant.

## Data Availability

De-identified data underlying the findings are available from the corresponding author upon reasonable request.
